# Do self-reported pregnancy complications add to risk evaluation in older women with established cardiovascular disease?

**DOI:** 10.1186/s12905-019-0851-x

**Published:** 2019-12-16

**Authors:** Elin Täufer Cederlöf, Nina Johnston, Jerzy Leppert, Pär Hedberg, Bertil Lindahl, Christina Christersson

**Affiliations:** 1 Department of Medical Sciences, Cardiology, Uppsala University, S-751 85 Uppsala, Sweden; 2 Centre for Clinical Research, Uppsala University, Västmanland County Hospital, Västerås, Sweden; 3 Department of Clinical Physiology, Uppsala University, Västmanland County Hospital, Västerås, Sweden; 4 Uppsala Clinical Research Center, Uppsala University, Uppsala, Sweden

**Keywords:** Pregnancy complications, Atherosclerosis, Cardiovascular disease

## Abstract

**Background:**

In postmenopausal women with established cardiovascular disease (CVD), it is unknown whether a history of pregnancy complications are related to multisite artery disease (MSAD), defined as atherosclerotic lesions in at least two major vascular beds. Pregnancy complications are an established risk factor for CVD. This study aimed to investigate the frequency of pregnancy complications and their association to specific atherosclerotic manifestations and prediction of MSAD in older women with and without CVD.

**Methods:**

In total, 556 women were invited to participate in the study. Of these women 307 reported former pregnancy from a cohort of women with (*n* = 233) and without CVD (*n* = 74). The self-reported frequency of pregnancy complications were surveyed retrospectively by a questionnaire that included miscarriage, subfertility, gestational hypertension (GHT) and/or preeclampsia (PE), low birth weight, preterm birth, bleeding in late pregnancy, gestational diabetes mellitus and high birth weight. Three vascular beds were examined, the peripheral, carotid and coronary arteries.

**Results:**

The mean age was 67.5 (SD 9.5) years. GHT and/or PE tended to be more common, but not significant, in women with CVD than in women without (20.3% vs 10.8%, *p* = 0.066). Among women with GHT and/or PE, hypertension later in life were more frequent than in women without (66.7% vs 47.4%, *p* = 0.010). GHT and/or PE were not associated with specific atherosclerotic manifestations or prediction of MSAD.

**Conclusions:**

In older women with established CVD, pregnancy complications was not associated to specific atherosclerotic manifestations and may not provide additional value to the risk evaluation for MSAD.

## Background

In postmenopausal women with established cardiovascular disease (CVD), it is unknown whether a history of pregnancy complications are associated to specific atherosclerotic manifestations or is associated to prediction of multisite artery disease (MSAD), defined as atherosclerotic lesions in at least two major vascular beds [1]. The importance of pregnancy complications in relation to other traditional risk factors for CVD is also unknown. It is well described that several pregnancy complications increase the risk for CVD [2, 3], as well as the menopause, age and other traditional risk factors. CVD and pregnancy complications likely share possible pathophysiological mechanisms in terms of vascular function, immunoregulation and metabolic control [4]. Some pregnancy complications have been associated with an earlier development of traditional risk factors [5].

The association between common pregnancy complications and future risk of CVD has received attention in the latest CVD prevention guidelines that highlighted gestational hypertension (GHT), preeclampsia (PE), and gestational diabetes mellitus (GDM) [2, 3, 6]. Other pregnancy complications that had been suggested to increase the risk of CVD are miscarriage, subfertility, low birth weight (LBW), preterm birth, bleeding in late pregnancy and high birth weight (HBW) [7–11]. Most studies of pregnancy complications and risk of CVD studied younger women without symptoms of disease [12, 13].

CVD mostly occur at an older age. A high proportion of postmenopausal women have findings of asymptomatic atherosclerosis at examination and traditional risk factors increase this risk [14, 15]. Atherosclerotic involvement in one vascular bed also increases the risk of engagement in another. When present with the first clinical manifestation of CVD, findings of asymptomatic atherosclerosis at examination of the vessels are common. The total burden of atherosclerosis is associated to increased risk of future CVD events and worse prognosis [1, 16]. The knowledge about the association of pregnancy complications and atherosclerosis in patients with established CVD is limited.

Although studies report conflicting findings regarding the reliability of self-reported pregnancy history, self-reporting is the most common means of obtaining this information especially in older women for which other types of documentation may not be available [17, 18]. The study aimed to investigate whether self-reported pregnancy complications were more common in postmenopausal women with CVD than without. The aim of the study was also to determine whether self-reported pregnancy complications could be associated to specific manifestations of atherosclerosis and whether they could predict MSAD in older women.

## Methods

### Study population

**Fig. 1:**
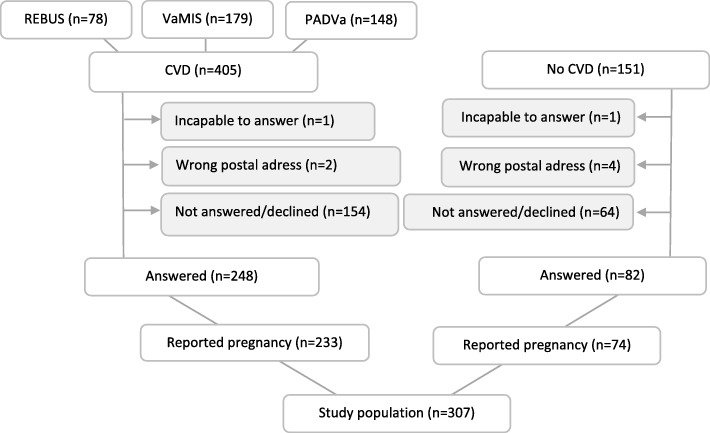
Description of the study population. *REBUS* RElevance of Biomarkers for future risk of thromboembolic events in UnSelected post-myocardial infarction patients, *VaMIS* Västmanland Myocardial Infarction Study, *PADVa* Peripheral Artery Disease in Västmanland, *CVD* Cardiovascular disease

Study participants with CVD (myocardial infarction (MI) or peripheral artery disease (PAD)) were recruited from three patient cohorts; REBUS (RElevance of Biomarkers for future risk of thromboembolic events in UnSelected post-myocardial infarction patients) [19], VaMIS (Västmanland Myocardial Infarction Study) [20] and PADVa (Peripheral Artery Disease in Västmanland) [21]. Study participants without CVD were recruited from controls of the VaMIS study. In total 556 women were included in the present study (Fig. 1).

### Description of the cohorts

The REBUS study included patients with MI according to the universal definition [22] at the Department of Cardiology at Uppsala University Hospital in 2010–2012 [19]. Exclusion criteria were death within five days of MI or inability to participate in follow up and the cohort represents an unselected MI population.

The VaMIS study included patients with MI according to the universal definition [20]. Exclusion criteria were dementia, acute confusion, and other severe disease or language difficulties.

In the PADVa study, consecutive patients referred to the Vascular Ultrasound Laboratory of the department of Vascular Surgery at Västmanland County Hospital were evaluated for inclusion [21]. Inclusion criteria was at least one of the following; mild to severe stenosis of the internal carotid artery, symptoms of claudication combined with an ankle brachial index (ABI) ≤0.90 or patients with symptoms of claudication combined with ultrasonographic evidence of arterial occlusive disease in the ipsilateral extremity. There were no exclusion criteria in the PADVa study. The VaMIS and PADVa studies were conducted at Västmanland County Hospital in Västerås in 2005–2011.

The cohort without CVD was recruited from the control group of VaMIS. For each VaMIS patient included, a control subject was recruited from the general population by use of the Swedish Population Register, were all Swedish citizens are registered. General population citizens with the closest date of birth, same sex and same municipality as the VaMIS patient were invited to participate by mail [21]. Women with atherosclerotic manifestations at examination were excluded.

In all cohorts, baseline demographics were collected at inclusion. Risk factors included body mass index (BMI) and smoking. Smoking status was subdivided into current or former/never. Information about education and income were not available. Co-morbidities included hypertension and diabetes mellitus, both medically treated and journal verified, and previous clinical diagnosis of MI, stroke, transient ischemic attack or PAD.

### Methods for assessment of atherosclerosis

The prevalence of atherosclerosis was assessed in three vascular beds (peripheral, carotid and coronary arteries) with methods clinically applicable.

Of the women reporting a former pregnancy 96.1% (*n* = 295) underwent examination of the peripheral arteries by measuring ABI at an outpatient visit. PAD was defined as an abnormal ABI score (< 0.9 or > 1.4) in any one measurement and defined an abnormal finding of the peripheral arteries [23].

Of the women reporting a former pregnancy 97.7% (*n* = 300) underwent carotid ultrasonography. The carotid arteries were assessed using a two-dimensional Doppler ultrasound examination of the internal carotid artery or the common carotid artery. Colour duplex and flow velocities were used to grade stenosis. Atherosclerosis or a previous history of carotid endarterectomy defined an abnormal finding of the carotid arteries [24].

Coronary angiography was done during hospital admission when clinically indicated. Of the women that reported former pregnancy, 137 women underwent coronary angiography, 96.6% (*n* = 57) of the patients in REBUS and 95.2% (*n* = 80) of the patients in VaMIS. None of the women in PADVa or in the cohort without CVD underwent coronary angiography. The findings at coronary angiography were classified as normal (0–29%), non-significant stenosis (30–50%), or significant stenosis (> 50%), and/or occlusion. A stenosis of 30% or more defined an abnormal finding of the coronary arteries [25]. MSAD was defined as at least two abnormal findings of the vascular beds examined.

### Description of the questionnaire of self-reported pregnancy complications

The questionnaire was based on questions proposed by Roberts and Hubel [26] and included the following pregnancy complications: a history of miscarriage, subfertility, GHT and/or PE, LBW, preterm birth, bleeding in late pregnancy, GDM and HBW (definitions are found in the Additional file 1). In total 8 women died before the questionnaire was sent and were excluded (Additional file 1). The questionnaire was sent to the participants in REBUS in 2013 and to the participants in VaMIS, PADVa and to the women without CVD in 2016. One reminder was sent to those who did not reply at first.

### Statistical analysis

For descriptive statistics, continuous variables were described as means and standard deviation (SD) and categorical variables as numbers and percentages. Comparison was done between women with and without CVD. The pregnancy complication GHT and/or PE were studied in separate for each of the studied atherosclerotic manifestations. Normally distributed variables were compared by independent *t*-test. Categorical variables were compared by chi-square test and Fisher’s exact test where appropriate. A *p*-value < 0.05 was considered statistically significant.

Univariate and multivariate associations between outcome and risk factors were examined with logistic regression models and were presented as estimated odds ratios (OR) with 95% confidence intervals (CI). The analysis included GHT and/or PE and outcome of MSAD. A separate analysis was performed of the women with MI (REBUS and VaMIS). In the multivariate model, adjustment was done for age and smoking. IBM® SPSS Statistics version 24 was used for all statistical analyses.

## Results

In total 556 women were invited to participate in the study by mail. The response rate to the questionnaire was 59.4% (*n* = 330) and more detailed information about the response rate in the different cohorts are found in the Additional file 1. The women who did answer the questionnaire were younger than those who did not (Additional file 1). There were minor differences in mean age between the three cohorts in women with CVD (Additional file 1). Of the 330 women who answered the questionnaire 93.0% (*n* = 307) reported at least one former pregnancy. Previous MI was more common in women who did not report former pregnancy than those who did (Additional file 1). The present study cohort consisted of 307 women who reported a former pregnancy, including women with CVD and without.

### Baseline characteristics

The mean age of the 307 women who reported a former pregnancy was 67.5 (SD 9.5) years. In women with CVD the frequency of current smokers was 23.7% (*n* = 55) compared with 10.8% (*n* = 8) of the women without CVD (*p* = 0.017) (Table 1). There was a higher frequency of comorbidities in women with CVD than in women without (Table 1).

**Table 1:** Baseline characteristics, women with CVD versus without (*n* = 307)

	CVD (*n* = 233)	No CVD (*n* = 74)	Missing	*p*-value
Covariates				
Age at inclusion	68.5 (9.2)	64.3 (9.8)		< 0.001*
BMI	27.0 (4.4)	26.2 (4.6)		0.2*
Hypertension	142 (60.9)	13 (17.6)		< 0.001**
Current smoker	55 (23.7)	8 (10.8)	1	0.017**
Diabetes mellitus	36 (15.5)	2 (2.7)		0.004**
Previous stroke or TIA	8 (3.4)	0 (0)		0.2**
Previous MI	21 (9.0)	0 (0)		0.007**
Previous PAD	34 (14.6)	0 (0)		< 0.001**
Pregnancy complications				
Miscarriage	51 (22.4)	16 (21.6)	5	0.9**
Subfertility	41 (18.6)	11 (15.5)	16	0.5**
GHT and/or PE	46 (20.3)	8 (10.8)	6	0.066**
LBW (< 2500 g)	21 (9.5)	7 (9.5)	11	1.0**
Preterm birth (< 37 weeks)	30 (14.2)	6 (8.1)	21	0.2**
Bleeding in late pregnancy	9 (3.9)	2 (2.7)	5	1.0**
GDM	3 (1.3)	2 (2.7)	5	0.6**
HBW (> 4500 g)	12 (5.4)	4 (5.4)	11	1.0**

Values are described as means (SD = standard deviation) for numerical variables and *n* (%) for categorical variables

*CVD* Cardiovascular disease, *BMI* Body Mass Index, *TIA* Transient ischemic attack, *MI* Myocardial infarction, *PAD* Peripheral artery disease, *GHT* Gestational hypertension, *PE* Preeclampsia, *LBW* Low birth weight, *GDM* Gestational diabetes mellitus, *HBW* High birth weight

*p-value by Independent *t*-test, **p-value by Chi-square test or Fisher’s exact test where appropriate

### Manifestation of atherosclerosis

In women with CVD, 25.1% (*n* = 74) had abnormal ABI and 48.3% (*n* = 145) had atherosclerosis at carotid duplex examination. Out of the 137 who underwent coronary angiography, 94.1% (*n* = 129) had an abnormal finding. In the coronary arteries non-significant stenosis was found in 8.7% (n = 12), single vessel disease in 52.6% (*n* = 72) and double or triple coronary vessel disease in 32.8% (*n* = 45).

### Self-reported pregnancy complications

The mean parity of women with CVD was 2.49 (SD 1.1) compared to 2.47 (SD 0.9) in women without. In women with CVD 25.9% (*n* = 59) reported more than one pregnancy complication compared with 19.2% (*n* = 14) of women without (*p* = 0.2). Miscarriage and subfertility were frequently reported but without differences between the groups (Table 1).

A higher frequency, but not significant, was found for GHT and/or PE in women with CVD compared with women without, 20.3% (*n* = 46) versus 10.8% (*n* = 8) (*p* = 0.066) (Table 1). In a logistic univariate model, there was a trend towards an association of GHT and/or PE and CVD (OR 2.10; CI 0.93–4.61; *p* = 0.077) which was slightly strengthened after adjustment for age and smoking (OR 2.28; CI 1.00–5.22; *p* = 0.051). The frequency of other self-reported pregnancy complications did not differ among women with CVD or without (Table 1). Few women reported GDM without difference between women with CVD or without (Table 1). The frequency of pregnancy complications in the separate patient cohorts of women with CVD are found in the Additional file 1.

Hypertension later in life was more common in women with a history of GHT and/or PE than without, 66.7% versus 47.4% (*p* = 0.010). There was no association between smoking later in life and a history of GHT and/or PE. Diabetes mellitus later in life tended to be more common in women with a history of GHT and/or PE than without, 20.4% (*n* = 11) of the women with GHT and/or PE had diabetes mellitus later in life compared to 10.9% (*n* = 27) without (*p* = 0.059).

### Self-reported pregnancy complications and manifestations of atherosclerosis

GHT and/or PE were not associated with abnormal ABI, abnormal findings of the carotids or with abnormal findings in both locations (Table 2). Neither GHT and/or PE were associated with abnormal findings of the coronary arteries (Table 2). In women who reported bleeding in late pregnancy, an abnormal ABI was found in 63.6% (*n* = 7) compared with 23.3% (*n* = 65) of the women without (*p* = 0.006).

**Table 2:** GHT and/or PE and atherosclerotic manifestations

	GHT and/or PE		*p*-value**
	*Yes*	*No*	
ABI (*n* = 289*)	12 (23.1)	60 (25.3)	0.7
-*Abnormal*			
-*Normal*	40 (76.9)	177 (74.7)	
Carotid (*n* = 294*)	26 (51.0)	115 (47.3)	0.6
-*Abnormal*			
-*Normal*	25 (49.0)	128 (52.7)	
ABI and carotid (*n* = 286*)	10 (19.6)	47 (20.0)	0.9
-*Abnormal*			
-*Normal*	41 (80.4)	188 (80.0)	
Coronary (*n* = 134*)	5 (4.8)	3 (10.3)	0.4
-*Abnormal*			
-*Normal*	100 (95.2)	26 (89.7)	
Coronary and ABI or Carotid (*n* = 120*)	13 (21.3)	13 (22.0)	0.9
-*Abnormal*			
-*Normal*	48 (78.7)	46 (78.0)	

Values are described as *n* (%) of women with abnormal and normal findings in the separate vascular beds with and without GHT and/or PE

*ABI* Ankle brachial index, *GHT* Gestational hypertension, *PE* Preeclampsia

*Total number of women who underwent the examination and answered the question about GHT and PE. **p-value by Chi-square test or Fisher’s exact test when appropriate

### Self-reported pregnancy complications and manifestations of multisite artery disease

The self-reported pregnancy complications GHT and/or PE were evaluated for prediction of MSAD (Table 3). In a univariate logistic regression model GHT and/or PE was not associated with MSAD (OR of 0.74; CI 0.37–1.51; *p* = 0.4) nor after adjustment for age and smoking (OR 0.77; CI 0.38–1.58; *p* = 0.5) (Table 3). The same outcome was studied for patients with myocardial infarction (REBUS and VaMIS). GHT and/or PE had an OR of 0.75 (CI 0.33–1.71 *p* = 0.5) and persisted after adjustment for age and smoking 0.79 (CI 0.34–1.86 *p* = 0.6) (Table 3).

**Table 3:** GHT and/or PE and prediction of MSAD

Population	GHT and/or PE			
	Univariate	*p*-value	Multivariate	*p*-value
-In all patients	0.74 (0.37–1.51)	0.4	0.77 (0.38–1.58)	0.5
-In MI patients	0.75 (0.33–1.71)	0.5	0.79 (0.34–1.86)	0.6

Logistic regression model, adjusted for age and smoking. Values are described as OR (CI)

*GHT* Gestational hypertension, *PE* Preeclampsia, *MSAD* Multisite artery disease, *MI* Myocardial infarction

## Discussion

Pregnancy complications were frequently reported in both women with CVD and without. GHT and/or PE tended to be more common, but not significant, in women with CVD than without. Pregnancy complications were not associated with specific manifestations of atherosclerosis and did not predict MSAD in women with CVD.

Pregnancy complications have been described to be associated with an earlier development of traditional risk factors [5]. GHT and/or PE was the only pregnancy complication that tended to differ between the groups and this finding was likely due to an enrichment of the complication rate in a population with a high proportion of hypertension. In women who reported GHT and/or PE, hypertension later in life was more common. Diabetes mellitus later in life also tended to be more common in women with a history of GHT and/or PE. These associations have been described elsewhere [27] and can be a mediating factor to the risk of CVD. The same relationship between GDM and later manifestation of diabetes mellitus have also been published [27] but was nothing we could confirm due to unexpectedly few cases of GDM. An explanation for this could be a difference in the threshold for diagnosing diabetes mellitus when the participants were in their childbearing age [28].

No associations between pregnancy complications and manifestations of atherosclerosis were found and pregnancy complications did not predict MSAD in women with CVD. Our findings are supported by a recent population-based, prospective cohort study, were pregnancy complications (PE, GHT, preterm birth and small for gestational age) led to only small improvements in CVD prediction. Pregnancy complications were not strong enough independent predictors of CVD after adjusting for established risk factors [29].

Pregnancy complications might be associated with atherosclerosis through many different pathophysiological mechanisms (e.g. endothelial dysfunction, inflammation, metabolic derangements) and/or coincidental cardiovascular risk factors [30]. PE may accelerate the progress of atherosclerosis [31]. In a recent study, a history of hypertension in pregnancy was associated with later development of specifically PAD [32] but we could not confirm these findings. We found a significant and previously not described association between bleeding in late pregnancy and PAD. However, this finding must be interpreted with great caution due to the low number of cases and needs to be confirmed in future studies. A common cause of antepartum bleeding in late pregnancy is placental abruption, which has been associated with increased cardiovascular mortality [33]. At retrospective assessment, miscarriage was the most frequent complication reported and the frequency was in accordance with other studies [34]. Miscarriage has been found to be associated with a higher risk for coronary heart disease in a recent meta-analysis [35]. Subfertility was also common and was reported in a slightly higher frequency than numbers published elsewhere [36].

Earlier studies that concluded associations between different pregnancy complications and risk of CVD mostly studied younger women [12, 13] even if there are some that also included older [37]. Others have reported that the risk for CVD for pregnancy complications seems to decline with age [12, 38]. In contrast to others, we included older postmenopausal women with symptoms of disease and compared them to women without CVD regarding atherosclerotic manifestations. Our results suggest that in older women, traditional risk factors for CVD are important in the risk evaluation of atherosclerosis whereas pregnancy complications have less value.

### Limitations

Limitations to this study include the small number of participants both in the group with CVD and without and that restricted the numbers of possible confounders to adjust for in the multivariate analysis. No information of education and income were available. Women with atherosclerotic manifestations were excluded from the control group to simplify the comparability. Pregnancy history was collected retrospectively and as such, the information gathered is subject to recall- and misclassification bias. Another limitation was the relative low and varying response rate in the separate cohorts of the women with CVD. However, the response rates was as expected since the response rates of questionnaire-based studies in general have dropped dramatically in the last decades [39].

## Conclusions

With the limitation of the small sample size of the present study, we found pregnancy complications to be common in both women with CVD and without. Pregnancy complications were not associated with atherosclerotic manifestations and did not predict MSAD. In older postmenopausal women with manifest CVD, pregnancy complications may not provide additional value to the risk evaluation for the extent of atherosclerosis when other traditional risk factors for CVD are present but this has to be confirmed in larger longitudinal studies with verified diagnoses.

## Supplementary information

**Additional file 1.** Supplementary information.

## Data Availability

The datasets generated and analyzed during the current study are not publicly available due to individual privacy of the study participants.

## Abbreviations

ABI: Ankle brachial index
BMI: Body mass index
CI: Confidence interval
CVD: Cardiovascular disease
GDM: Gestational diabetes mellitus
GHT: Gestational hypertension
HBW: High birth weight
LBW: Low birth weight
MI: Myocardial infarction
MSAD: Multisite artery disease
OR: Odds ratio
PAD: Peripheral artery disease
PADVa: Peripheral Artery Disease in Västmanland
PE: Preeclampsia
REBUS: RElevance of Biomarkers for future risk of thromboembolic events in UnSelected post-myocardial infarction patients
SD: Standard deviation
VaMIS: Västmanland Myocardial Infarction Study
