# Supplementary material for: Do self-reported pregnancy complications add to risk evaluation in older women with established cardiovascular disease?
Source: BMC Womens Health. 2019 Dec 16;19:160. doi: 10.1186/s12905-019-0851-x (PMC6916002; doi:10.1186/s12905-019-0851-x)
Supplement: Supplementary file 1 — Additional file 1. Supplementary information. [file 12905_2019_851_MOESM1_ESM.pdf]

Supplementary material for

# Do self-reported pregnancy complications add to risk evaluation in older women with established cardiovascular disease?

Standard definitions and questions used to capture the different pregnancy complications

| Complication | Definition | Question posed |
|---|---|---|
| Miscarriage | Spontaneous abortion before 22 weeks of gestational age. | Did you experience a miscarriage during any pregnancy? |
| Subfertility | A duration of 1 year or more of involuntary childlessness. | Did you experience problems in attempts to get pregnant for a period of 1 year or longer? |
| Gestational hypertension | Blood pressure ≥ 140/90 at two times with 4-6 hours between measurements and after 20 weeks of gestational age with remission within 12 weeks postpartum. | Did you have high blood pressure during any pregnancy? |
| Preeclampsia | Blood pressure ≥ 140/90 at two times with 4-6 hours and proteinuria ≥ 0.3 g/24h after the 20th week of pregnancy. | Did you have preeclampsia during any pregnancy? |
| Low birth weight | Infant weight under 2500 g at the time of delivery. | What was the birth weight of your child/children? |
| Preterm birth | Delivery before 37 weeks of gestational age. | In what week of pregnancy was your child born (normal duration of a pregnancy is 40 weeks long)? (REBUS)<br><br>Did you deliver any child preterm, i.e. before 37 weeks of pregnancy? (Non-prevalent CVD group, VaMIS, PADVa) |
| Bleeding in late pregnancy | Bleeding in the last 6 months of pregnancy. | Did you experience any bleeding during the last 6 months of any pregnancy? |
| Gestational diabetes mellitus | High blood glucose during pregnancy without a previous diagnosis of diabetes mellitus and with remission within 12 weeks postpartum. | Did you have diabetes during any pregnancy? |
| High birth weight | Infant weight over 4500 g at the time of delivery. | What was the birth weight of your child/children? |

The questionnaire also included a question if any medications were taken during pregnancy for treatment of hypertension or diabetes.

Baseline characteristics, those answered the questionnaire compared to those not answered

| | Answered (n=330) | Not answered (n=226) | p-value |
|---|---|---|---|
| Age at inclusion | 67.6 (9.5) | 69.9 (9.7) | 0.005* |
| BMI | 26.8 (4.6) | 27.3 (5.5) | 0.2* |
| Hypertension | 178 (53.9) | 128 (56.6) | 0.5** |
| Current smoker | 64 (19.5) | 60 (26.5) | 0.049** |
| Diabetes mellitus | 41 (12.4) | 29 (12.8) | 0.9** |
| Previous stroke or TIA | 9 (2.7) | 7 (3.1) | 0.8** |
| Previous MI | 26 (7.9) | 14 (6.2) | 0.4** |
| Previous PAD | 35 (10.6) | 20 (8.8) | 0.5** |

Values are described as Means (SD=standard deviation) for numerical variables and n (%) for categorical variables. **p*-value by Independent *t*-test, ***p*-value by Chi-square test or Fisher´s exact test where appropriate.

*BMI* Body Mass Index, *TIA* Transient ischemic attack, *MI* Myocardial infarction, *PAD* Peripheral artery disease.

Baseline characteristics, those with earlier pregnancy compared to those without pregnancy

| | Reported pregnancy (n=307) | Reported not pregnancy (n=23) | p-value |
|---|---|---|---|
| Age at inclusion | 67.5 (9.5) | 68.6 (9.6) | 0.6* |
| BMI | 26.8 (4.4) | 27.1 (6.1) | 0.8* |
| Hypertension | 165 (53.7) | 15 (65.2) | 0.3** |
| Current smoker | 63 (20.6) | 1 (4.3) | 0.059** |
| Diabetes mellitus | 38 (12.4) | 3 (13.0) | 1.0** |
| Previous stroke or TIA | 9 (2.9) | 0 (0) | 1.0** |
| Previous MI | 21 (6.8) | 5 (21.7) | 0.026** |
| Previous PAD | 33 (10.7) | 1 (4.3) | 0.5** |

Values are described as Means (SD=standard deviation) for numerical variables and *n* (%) for categorical variables. **p*-value by Independent *t*-test, ***p*-value by Chi-square test or Fisher´s exact test where appropriate.

*BMI* Body Mass Index, *TIA* Transient ischemic attack, *MI* Myocardial infarction, *PAD* Peripheral artery disease.

Baseline characteristics, of the separate patient cohorts

| | REBUS (n=59) | VaMIS (n=84) | PADVa (n=90) |
|---|---|---|---|
| Age at inclusion | 69.1 (10.1) | 67.9 (10.1) | 68.7 (7.7) |
| BMI | 26.4 (4.0) | 27.5 (5.2) | 26.8 (3.8) |
| Hypertension | 32 (54.2) | 43 (51.2) | 67 (74.4) |
| Current smoker | 16 (27.1) | 23 (27.7)* | 16 (17.8) |
| Diabetes mellitus | 7 (11.9) | 10 (11.9) | 19 (21.1) |
| Previous stroke or TIA | 2 (3.4) | 1 (1.2) | 5 (5.6) |
| Previous MI | 5 (8.5) | 9 (10.7) | 7 (7.8) |
| Previous PAD | 2 (3.4) | 2 (2.4) | 30 (33.3) |

Values are described as Means (SD=standard deviation) for numerical variables and *n* (%) for categorical variables.

*BMI* Body Mass Index, *TIA* Transient ischemic attack, *MI* Myocardial infarction, *PAD* Peripheral artery disease. *$n$ = 83.

Baseline characteristics, of the deceased women before the questionnaire was sent

| | CVD (n=6)* | No CVD (n=2) |
|---|---|---|
| Age at inclusion | 69.5 (11.2) | 72.8 (5.2) |
| BMI | 27.3 (6.8) | 24.9 (4.9) |
| Hypertension | 5 (83.3) | 0 |
| Current smoker | 2 (33.3) | 0 |
| Diabetes mellitus | 1 (16.7) | 0 |
| Previous stroke or TIA | 0 | 0 |
| Previous MI | 2 (33.3) | 0 |
| Previous PAD | 2 (33.3) | 0 |

Values are described as Means (SD=standard deviation) for numerical variables and *n* (%) for categorical variables.

*CVD* Cardiovascular disease, *BMI* Body Mass Index, *TIA* Transient ischemic attack, *MI* Myocardial infarction, *PAD* Peripheral artery disease.

* 3 in REBUS, 1 in VaMIS, 2 in PADVa.

Pregnancy complications, in the separate patient cohorts

| | REBUS (n=59) | VaMIS (n=84) | PADVa (n=90) | Missing |
|---|---|---|---|---|
| Miscarriage | 14 (24.1) | 14 (17.1) | 23 (26.1) | 5 |
| Subfertility | 8 (13.8) | 16 (20.0) | 17 (20.7) | 13 |
| GHT and/or PE | 15 (25.9) | 17 (20.7) | 14 (16.1) | 6 |
| LBW (<2500 g) | 7 (12.3) | 7 (8.8) | 7 (8.2) | 11 |
| Preterm birth (<37 weeks) | 12 (30.0) | 8 (9.6) | 10 (11.2) | 21 |
| Bleeding in late pregnancy | 3 (5.2) | 2 (2.4) | 4 (4.5) | 5 |
| GDM | 0 (0) | 1 (1.2) | 2 (2.3) | 5 |
| HBW (>4500 g) | 3 (5.3) | 5 (6.3) | 4 (4.7) | 11 |

Values are described as Means (SD=standard deviation) for numerical variables and *n* (%) for categorical variables.

*GHT* Gestational hypertension, *PE* preeclampsia, *LBW* Low birth weight, *GDM* Gestational diabetes mellitus, *HBW* High birth weight.

Response rate, in the separate patient cohorts

| | CVD (n=405) | | | No CVD (n=151) | Total (n=556) |
|---|---|---|---|---|---|
| | REBUS (n=78) | VaMIS (n=179) | PADVa (n=148) | | |
| Response rate | 63 (80.8) | 93 (52.0) | 92 (62.2) | 82 (54.3) | 330 (59.4) |
| Former pregnancy | 59 (93.7) | 84 (90.3) | 90 (97.8) | 74 (49.0) | 307 (55.2) |

Values are described as answered questionnaires *n* (%).

*CVD* Cardiovascular disease

English version of the questionnaire developed for use in this study

**Instructions:**

The purpose of this questionnaire is to study heart and vessel health in women. There are two types of questions used. Some are answered by ticking a box and some are answered by the shortest amount of text. If you are unsure about your answer or of you do not want to answer leave the question blank.

**General questions:**

1. Have you stopped to menstruate?

   ☐ Yes, when was the last menstruation?_______ ☐ No ☐ I do not know
2. Have you ever been pregnant?

   ☐ Yes ☐ No
3. Have you ever had difficulties getting pregnant during at least one year?

   ☐ Yes ☐ No

**Pregnancy related questions:**

4. How many times have you been pregnant? ______________________________
5. Your age at your pregnancies: __________________________________ years
6. How many children have you been given birth to? ________________________
7. The sex(es) of your child(ren)? _______________________________________
8. The approximately birthweight of your child(ren) (round off to the closest weight in kilograms) _______________________________________________
9. <u>Version in REBUS:</u>

   In what week of pregnancy was your child born (normal duration of a pregnancy is 40 weeks long)?

   <u>Version in VaMIS, PADVa and women without CVD:</u>

   Were any of your children born preterm, before week 37 of pregnancy? (the normal length of a pregnancy is 40 weeks)

   ☐ Yes ☐ No

   If the answer was “yes”. Were any of your children born very preterm, before week 34 of pregnancy?

   ☐ Yes ☐ No

10. Did you breastfeed any of your children during at least one month?

☐ Yes ☐ No

11. In how many month of your life have you approximately been breastfeeding, if you sum the total time of breastfeeding for your children?

_______________________________________________________month

12. Have you been suffering from any of the following conditions during or in close connection to a pregnancy?

☐ High blood pressure

☐ Diabetes

☐ Heart failure

☐ Heart attack

☐ Blood clot (leg, lung, brain)

☐ Preeclampsia

☐ Bleeding during the last 6 month of the pregnancy?

☐ Miscarriage

Other complication: _______________________________________

13. Did you take any medication during your pregnancy/ies, and if yes what did you take?

_____________________________________________________________
